# Granulocytic MDSC with Deficient CCR5 Alleviates Lipogenesis and Inflammation in Nonalcoholic Fatty Liver Disease

**DOI:** 10.3390/ijms232113048

**Published:** 2022-10-27

**Authors:** Tzu-Chieh Liao, Jiung-Pang Huang, Yu-Ting Tsai, Wei-Ching Shih, Chi-Chang Juan, Po-Shiuan Hsieh, Li-Man Hung, Chao-Lan Yu

**Affiliations:** 1 Department and Graduate Institute of Biomedical Sciences, and Healthy Aging Research Center, College of Medicine, Chang Gung University, Taoyuan 33302, Taiwan; 2 Department and Institute of Physiology, National Yang Ming Chiao Tung University, Taipei 11221, Taiwan; 3 Department of Physiology and Biophysics, and Graduate Institute of Medical Science, National Defense Medical Center, Taipei 11490, Taiwan; 4 Kidney Research Center, Chang Gung Memorial Hospital, Linkou, Taoyuan 33305, Taiwan; 5 Department of Nephrology, Chang Gung Memorial Hospital, Linkou, Taoyuan 33305, Taiwan

**Keywords:** CCR5, g-MDSC, NAFLD, STAT3, lipid metabolism, inflammation

## Abstract

C-C chemokine receptor type 5 (CCR5) positively contributes to the pathogenesis of nonalcoholic fatty liver disease (NAFLD), a common metabolic liver disease associated with chronic inflammation. CCR5 signaling also facilitates the immunosuppressive activity of a group of immature myeloid cells known as granulocytic myeloid-derived suppressor cells (g-MDSCs). While both hepatocyte and g-MDSC express CCR5, how CCR5 coordinates these two distinct cell types in the hepatic microenvironment remains largely unknown. Here, we used in vivo and ex vivo approaches to define the molecular details of how CCR5 mediates the crosstalk between hepatocytes and g-MDSCs in a mouse model of NAFLD. Global CCR5-deficient mice exhibited more severe steatosis, increased hepatic gene expression of lipogenesis, and exacerbated liver damage in diet-induced obesity. Either NAFLD or CCR5-deficiency per se is causative for the increase of g-MDSCs. Purified g-MDSCs have a higher survival rate in the fatty liver microenvironment, and blockade of CCR5 significantly decreases g-MDSCs’ expression of anti-inflammatory factors. On the other hand, the null of CCR5 signaling increases hepatocytes’ expression of lipogenic genes in the NAFLD microenvironment. Most importantly, inhibiting g-MDSCs’ CCR5 signaling in the fatty liver microenvironment dramatically reduces STAT3 signaling, lipogenic, and pro-inflammatory gene expression in primary hepatocytes. Adoptive cell transfer experiments further demonstrate that CCR5-deficient g-MDSCs mitigate hepatic lipogenic gene expression without facilitating pro-inflammatory cytokine production and liver damage in NAFLD mice. These results suggest that targeting g-MDSCs’ CCR5 signaling might serve as a potential therapeutic strategy for NAFLD.

## 1. Introduction

Nonalcoholic fatty liver disease (NAFLD) is a common liver disorder at present due to the typical western-style diet and sedentary lifestyle. The prevalence of NAFLD has been estimated to reach 25% of the global population [1,2]. NAFLD refers to a group of conditions that emanates from excess lipid accumulation in the liver, also known as steatosis, on account of metabolic disorders including obesity and dysregulated hepatic glucose metabolism. Without proper intervention, hepatic steatosis will progress into nonalcoholic steatohepatitis (NASH) or even liver cirrhosis [3]. Although studies in NAFLD are continuously active, current knowledge on the complex crosstalk between different hepatic cell types is limited.

C-C chemokine receptors hold a growing body of evidence in modulating the development of NAFLD by virtue of their importance in immune regulation [4,5]. Among those, C-C chemokine receptor type 5 (CCR5) signaling has been implicated in NAFLD for both parenchymal cells and non-parenchymal cells in the liver [6,7,8]. Specifically, CCR5 has been shown to be expressed in both mouse primary hepatocytes and human hepatocyte cell lines [9,10]. CCR5 is implicated in different mouse models of chronic liver diseases [11,12]; however, its contribution to NAFLD progression is less well-defined. Maraviroc, a CCR5 antagonist, could reverse hepatic steatosis, and reduce hepatic triglyceride content in obesity-induced NAFLD mouse models under a high-fat diet (HFD) [13,14]. In contrast, the recent literature on mouse alcoholic fatty liver disease showed the protective effect of CCR5 against hepatic inflammation [15]. These conflicting reports demonstrate the complexity of CCR5 regulation in the liver and suggest that CCR5 may serve as a double-bladed sword in the hepatic microenvironment targeting different cell types.

Myeloid-derived suppressor cells (MDSCs) are a heterogeneous pool of bone marrow-derived cells that consists of myeloid progenitor and immature myeloid cells with the potential of inhibiting T cell responses [16,17,18]. In mice, MDSCs are defined by co-expression of myeloid lineage differentiation antigens Gr-1 and CD11b. Murine MDSCs are mainly comprised of two subsets of population: granulocytic MDSCs (g-MDSCs; Ly-6G^hi^Ly-6C^int^) and monocytic MDSCs (m-MDSCs; Ly-6G^-^Ly-6C^hi^) [19]. Although both g-MDSCs and m-MDSCs suppress T-cell responses by changing L-arginine metabolism and secreting immune-suppressive cytokines in the microenvironment, their suppressive activities are carried out by distinct effector molecules and signaling pathways [20,21]. Functionally, g-MDSCs generate more reactive oxygen species and arginase-1 (ARG1). Early studies show that the signal transducer and activator of transcription 3 (STAT3) signaling promote mouse MDSC accumulation and activity by facilitating its proliferation and expression of proteins with key functions, respectively [22,23,24].

Notably, CCR5 signaling has been shown as a key player in the development of myeloid lineage. Increasing evidence suggests that signaling from CCR5 and its ligand, C-C motif chemokine ligand 5 (CCL5), determines the immunosuppressive phenotypes of a huge proportion of immature myeloid cells, including MDSCs, at tumor-bearing sites [25]. Additionally, the STAT3 activator, interleukin-6 (IL-6), was proven to induce CCR5 expression and strong immunosuppressive activity in MDSCs [26]. For g-MDSCs, CCR5 signaling is not only crucial for their mobilization but also pivotal for promoting their proliferation and potentiating their immunosuppressive activities at the tumor site [25,27,28]. Although the importance of the CCR5 signaling for MDSCs has been unveiled in the tumor microenvironment, its role in NAFLD has yet to be fully discovered. In this study, we aimed to understand the role of CCR5 signaling in the crosstalk between g-MDSCs and hepatocytes under NAFLD.

## 2. Results

### 2.1. Global Deficiency of CCR5 Exacerbates Liver Damage and Steatosis in NAFLD

**Figure 1:**
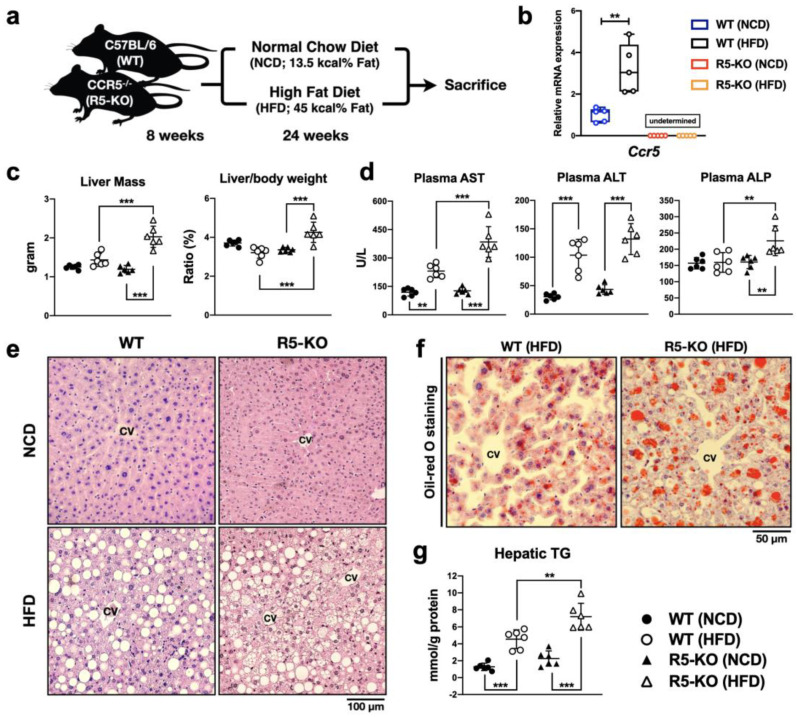
Global CCR5 deficiency exacerbates liver damage and steatosis in NAFLD. (**a**) A schematic diagram of animal and dietary model. (**b**) Hepatic Ccr5 mRNA level was detected by real-time PCR. Relative expression was normalized to *36b4* reference gene (n = 4–6). (**c**) Individual liver mass and body weight were measured and the ratio was calculated (n = 6). (**d**) Plasma concentrations of AST, ALT, and ALP were determined (n = 6). (**e**) Representative liver section images of hematoxylin and eosin staining. Positions of central vein (cv) are labeled. (**f**) Representative liver section images of Oil-red O staining. (**g**) Hepatic triglyceride concentrations were determined by colorimetric assay (n = 6). Representative and quantified results (means ± SD) are shown for the indicated number of mice. **, *p* < 0.005; ***, *p* < 0.001.

Previous studies suggest that CCR5 modulates liver functions and contributes to the pathogenesis of NAFLD. However, its detailed mechanism remains unclear. To dissect the role of CCR5 in hepatic metabolism, C57BL/6 wild-type (WT) and global CCR5 knockout (R5-KO) mice fed with normal chow diet (NCD) or HFD for 24 weeks were analyzed (Figure 1a). As expected, the transcript level of CCR5 was undetectable in both lean and obese R5-KO mice (Figure 1b). Furthermore, significant up-regulation of hepatic CCR5 expression was detected in HFD-induced NAFLD in WT mice (Figure 1b). Surprisingly, CCR5 deficiency remarkably enhanced liver mass and the liver-to-body weight ratio in obese mice (Figure 1c). To determine the extent of liver damage, we examined the levels of plasma aspartate aminotransferase (AST), alanine aminotransferase (ALT), and alkaline phosphatase (ALP), three well-known biomarkers. As shown in Figure 1d, all three biomarkers were elevated by HFD-induced obesity, except ALP in WT mice. More importantly, the levels of AST and ALP were significantly elevated in obese R5-KO mice as compared to obese WT mice, indicating that CCR5 signaling might avert liver damage from hepatic lipid deposition.

The extent of liver damage and steatosis was further visualized by histological analysis of liver sections. A significant increase in microvesicular steatosis was detected nearby the hepatic central vein in the R5-KO fatty liver (Figure 1e). Oil-red O staining for neutral triglyceride and lipid on frozen liver sections also showed higher intensity of signals around the hepatic central vein in the R5-KO fatty liver (Figure 1f). Consistent with a more acute form of lipid accumulation, R5-KO fatty liver exhibited significantly more triglyceride accumulation as compared to WT fatty liver (Figure 1g). Altogether, these results suggest that global CCR5 deficiency exacerbates liver steatosis and liver injury in obese mice.

### 2.2. Global Deficiency of CCR5 Elevates Hepatic g-MDSC in Both Lean and Obese Mice

**Figure 2:**
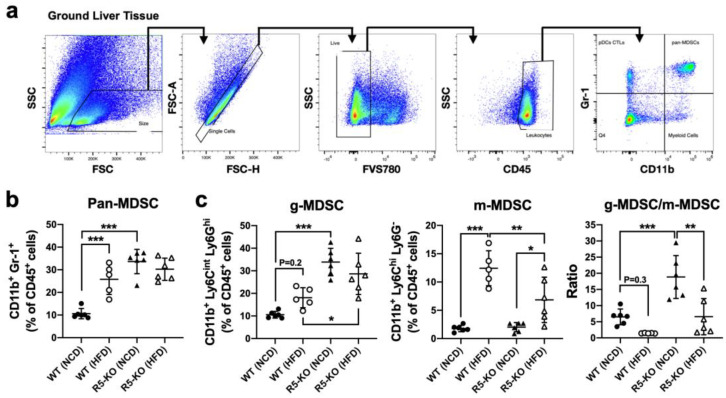
Global CCR5 deficiency elevates the hepatic g-MDSC population in both lean and obese mice. (**a**) Gating strategy for immunophenotyping hepatic MDSCs via flow cytometry. Percentages of hepatic MDSCs (**b**) and their subsets among the hematopoietic population (**c**) were distinguished. Representative and quantified results (means ± SD) are shown for the indicated number of mice. *, *p* < 0.05; **, *p* < 0.005; ***, *p* < 0.001.

Hepatic infiltration of a distinct population of immune cells and the subsequent inflammatory response plays an important role in liver damage associated with NAFLD. CCR5 is tightly connected with the immunological function of MDSCs, but the role of CCR5 in hepatic MDSCs is still unclear. We further immunophenotyped MDSC populations in the liver via flow cytometry. Hepatic leukocytes were gated on live single cells and the population of pan-MDSCs was determined by co-expression of CD45, CD11b, and Gr-1 surface markers (Figure 2a). In WT mice, the percentage of the pan-MDSC population among hepatic leukocytes was significantly elevated in NAFLD (Figure 2b). In lean mice, global deficiency of CCR5 alone was also sufficient to increase the percentage of hepatic pan-MDSCs, suggesting a pivotal role of CCR5 signaling in hepatic MDSC regulation. However, in obese mice, global CCR5 deficiency did not have a significant effect on the percentage of hepatic pan-MDSCs.

To further determine the sub-population of hepatic pan-MDSCs, surface staining of Gr-1 was substituted for staining of Ly-6C and Ly-6G in order to distinguish g-MDSC and m-MDSC. The result demonstrated an elevated percentage of hepatic g-MDSCs, although with a *p* value of 0.2, and m-MDSC in obesity-induced NAFLD of WT mice, as compared to their respective lean counterparts (Figure 2c). Most importantly, under both lean and obese conditions, CCR5 deficiency greatly increased the g-MDSC population among hepatic leukocytes (Figure 2c, left plot). It showed that the g-MDSC to m-MDSC ratio was remarkably susceptible to the absence of CCR5 (Figure 2c, right plot). Together, our data suggest that both NAFLD and CCR5 deficiency contribute to the elevation of the hepatic g-MDSC population.

### 2.3. Global Deficiency of CCR5 and HFD Both Activate Hepatic STAT3

**Figure 3:**
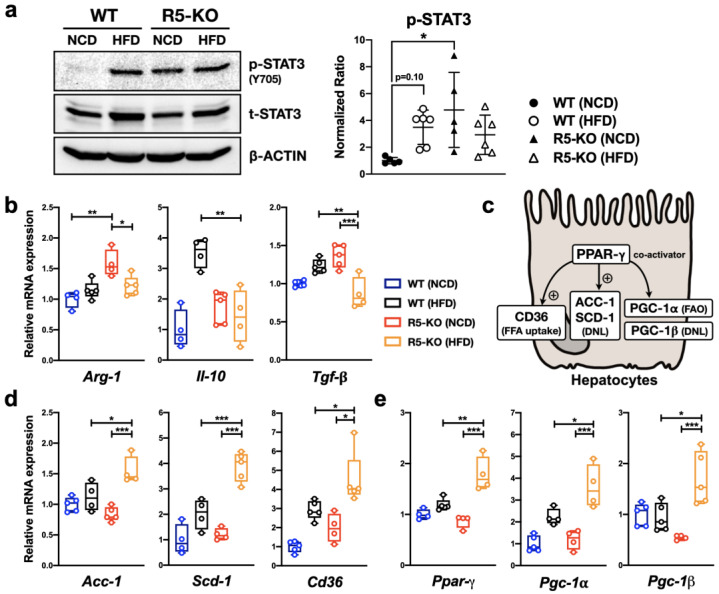
Global CCR5 deficiency causes altered hepatic STAT3 signaling and expression of cytokine genes and genes related to lipid metabolism. (**a**) Equal amounts of proteins from liver homogenates were subjected to SDS-PAGE followed by immunoblotting using antibodies specific for phosphorylated and total STAT3. Normalization was confirmed by β-ACTIN immunoblotting. Signal intensity was quantified for statistical analysis. The signal of phosphorylated STAT3 was normalized to total STAT3 and set the average value of WT (NCD) to 1. The levels of hepatic transcripts of genes related to g-MDSC functions (**b**), lipogenesis (**d**), and *Ppar-γ* with its co-activators (**e**) were determined by real-time PCR. Expression was normalized to the *36b4* reference gene and further normalized to the average value of the WT (NCD) group (n = 4-6). (**c**) A schematic diagram of targeted lipid-regulatory genes with their reported functions in NAFLD. FFA, free fatty acid; DNL, de novo lipogenesis; FAO, fatty acid oxidation. *, *p* < 0.05; **, *p* < 0.005; ***, *p* < 0.001.

As CCR5 deficiency leads to aberrant lipid metabolism (Figure 1) and altered immune profile (Figure 2) in the liver, it is very likely that CCR5 is heavily involved in regulating hepatic genes associated with lipid metabolism and inflammation. We, therefore, specified our target signaling pathways on STAT3 because of its critical roles in regulating the functional activity of g-MDSCs, modulating the hepatic immune response, and lipid accumulation [29]. STAT3 activation was determined by the level of phosphorylation at the key regulatory tyrosine residue through immunoblotting. As shown in Figure 3a, hepatic STAT3 was highly activated by HFD in WT mice with a *p* value of 0.1. Interestingly, CCR5 deficiency significantly triggered hepatic STAT3 phosphorylation in lean mice. It indicates that CCR5 has a distinct effect on STAT3 signaling in the hepatic microenvironment.

### 2.4. Global Deficiency of CCR5 Has Distinct Effects on Cytokine Gene Expression

The observation of STAT3 activation (Figure 3a) and increased population of g-MDSC (Figure 2) in the liver of lean R5-KO mice suggest an overall increase of g-MDSC activity in the hepatic microenvironment. To test this hypothesis, we examined the hepatic mRNA level of genes related to g-MDSC’s function in anti-inflammation, such as *Arg-1*, *Il-10*, and *Tgf-β*. Consistent with our hypothesis, the hepatic transcript levels of *Arg-1*, a STAT3-dependent gene critical for g-MDSC function, were significantly higher in the lean R5-KO mice (Figure 3b). On the other hand, the hepatic levels of *Il-10* and *Tgf-β*, other major anti-inflammatory cytokines secreted by g-MDSCs, were significantly lower in R5-KO obese mice compared with WT obese mice (Figure 3b). These findings suggest that CCR5 may modulate the anti-inflammatory function of hepatic g-MDSCs through both STAT3-dependent and STAT3-independent pathways.

### 2.5. Global Deficiency of CCR5 Synergizes with HFD in Regulating Lipid-Metabolic Genes

The exacerbated steatosis in obese R5-KO mice (Figure 1) suggests that CCR5 may also play a key role in hepatic lipid metabolism. In murine hepatocytes, *Ppar-γ* plays a steatogenic role in NAFLD by up-regulating the expression of *Cd36* involved in the uptake of free fatty acids (Figure 3c). Furthermore, *Ppar-γ* induces *Acc-1* and *Scd-1* expression which promotes de novo lipogenesis via intracellular triglyceride accumulation [30]. In addition, *Ppars*’ coactivator, *Pgc-1β*, blocks fatty acid β-oxidation while fueling up de novo lipogenesis to facilitate the progression of NAFLD. On the flip side, *Pgc-1α*, promotes fatty acid β-oxidation by enhancing mitochondrial biogenesis and reducing triglyceride storage in hepatocytes [31,32]. In our study, all of the aforementioned hepatic lipid-metabolic genes were dramatically increased in obese R5-KO mice compared with obese WT or lean R5-KO mice (Figure 3d,e). This solid evidence strongly supports the critical role of hepatic CCR5 in modulating lipid metabolism.

### 2.6. CCR5 Signaling Modulates Anti-Inflammatory Profile of g-MDSC in the Hepatic Microenvironment

**Figure 4:**
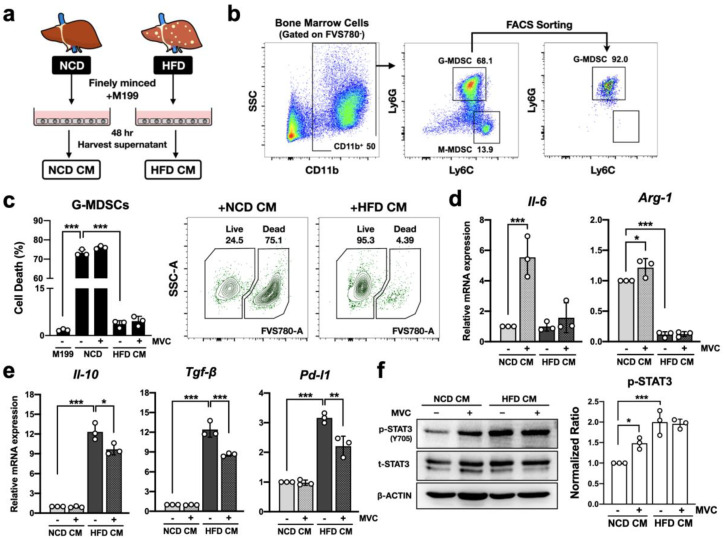
CCR5 signaling modulates the anti-inflammatory profile of g-MDSC in the hepatic microenvironment. (**a**) A schematic diagram of preparing liver-conditioned M199 culture medium (CM). (**b**) Primary g-MDSCs were isolated from WT bone marrow cells by FACS sorting. (**c**) Isolated g-MDSCs were pretreated with 5 μM Maraviroc (MVC) (+) or vehicle control (−) for 1 h and then stimulated with indicated liver-CM for 18 h. Cells were also treated with vehicle control and M199 medium as a negative control. Dead cells were quantitated by flow cytometry after fixable viability staining with FVS780. Additionally, g-MDSCs’ transcript levels of genes related to STAT3 activation (**d**) and anti-inflammation (**e**) were determined by real-time PCR. Expression was normalized to the *36b4* reference gene and further normalized to the average value of the first group (n = 3). (**f**) Primary WT g-MDSCs were pretreated with 5 μM MVC (+) or vehicle control (−) for 1 h and then stimulated with indicated liver-CM for 10 min. The levels of phosphorylated and total STAT3 were determined by immunoblotting. The signal of phosphorylated STAT3 was normalized to total STAT3 and set the first group as 1 (n = 3). *, *p* < 0.05; **, *p* < 0.005; ***, *p* < 0.001.

Given that both NAFLD and CCR5 deficiency significantly elevated the g-MDSC population among hepatic CD45^+^ leukocytes (Figure 2c), it becomes exceedingly interesting to define the effects of NAFLD microenvironment and CCR5 signaling on g-MDSCs. In order to study the molecular functions of g-MDSCs in response to NAFLD ex vivo, we generated a conditioned medium (CM) from culturing lean and obese WT mouse liver tissues (Figure 4a) to emulate the hepatic microenvironment. Primary g-MDSCs were isolated from WT bone marrow cells by fluorescence-activated cell sorting (FACS) (Figure 4b) and then exposed to NCD-CM versus HFD-CM for 18 h. As shown in Figure 4c, g-MDSCs have a significantly higher survival rate in the NAFLD microenvironment. However, pretreatment with the CCR5 antagonist Maraviroc did not affect the survival rate of g-MDSCs (Figure 4c).

To further dissect the molecular details of g-MDSC alteration, transcript levels of specific target genes in g-MDSCs were analyzed. As shown in Figure 4d, the expression of *Il-6*, a key cytokine to activate STAT3, was elevated by Maraviroc treatment with NCD-CM. The same pattern was observed in the expression of *Arg-1*, a STAT3-target gene critical for g-MDSC functions. Specifically, *Arg-1* gene expression was significantly reduced in the HFD liver microenvironment regardless of the treatment of Maraviroc. On the flip side, other pivotal anti-inflammatory genes, such as *Il-10*, *Tgf-β*, and *Pd-l1*, were up-regulated in the HFD liver microenvironment (Figure 4e). This up-regulation could be significantly reduced by the treatment of Maraviroc. Notably, Tgf-β has been suggested to be extremely important in facilitating immunosuppressive functions of g-MDSC. The expression pattern of *Tgf-β* in g-MDSC positively correlates with that in the whole liver tissue under the circumstance of NAFLD or CCR5-KO (Figure 3b). Although other types of cells, such as regulatory T cells and hepatic stellate cells, also produce Tgf-β in the liver [33,34], our result implies that g-MDSC may be one of the major contributors to Tgf-β production in the hepatic environment.

Moreover, short-term CM stimulation revealed that blockade of CCR5 signaling enhanced g-MDSCs’ STAT3 activation in a healthy liver environment but did not alter STAT3 activity in the NAFLD microenvironment (Figure 4f). These results are consistent with the expression of functional CCR5 on g-MDSCs [27]; however, they also suggest that CCR5 signaling may exhibit dual regulatory roles toward hepatic g-MDSCs. CCR5 may inhibit g-MDSCs’ anti-inflammatory function through STAT3-dependent regulation in healthy liver but facilitate one via STAT3-independent pathway in fatty liver.

### 2.7. CCR5 Regulates STAT3 Signaling Differently in Primary Hepatocytes

**Figure 5:**
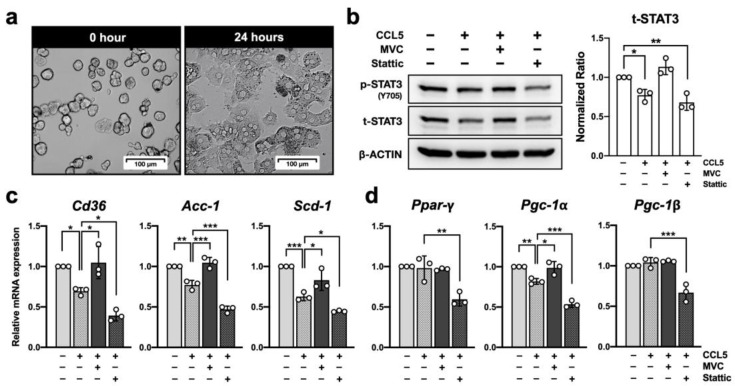
CCR5 regulates STAT3 signaling in primary hepatocytes. (**a**) Microscopy images of primary WT hepatocytes were taken before and after plating on a collagen-coated culture dish for 24 h. Subsequent experiments were performed in primary hepatocytes 24 h after plating. (**b**) Primary hepatocytes were pretreated with 5 μM MVC, 10 μM Stattic or vehicle control (−) for 1 h, and then either left unstimulated (−) or stimulated with 10 ng/mL of CCL5 for 18 h. Total lysates were analyzed by immunoblotting to determine the status of STAT3 expression and phosphorylation. The amount of total STAT3 was normalized to β-ACTIN and set the first group as 1 (n = 3). The levels of transcripts from genes related to hepatic lipogenesis (**c**) and *Ppar-γ* with its co-activators (**d**) were determined by real-time PCR. Expression was normalized to the *36b4* reference gene and further normalized to the average value of the first group (n = 3). *, *p* < 0.05; **, *p* < 0.005; ***, *p* < 0.001.

Considering that CCR5 deficiency affects hepatic STAT3 signaling and lipid metabolism (Figure 1 and Figure 3), it is essential to study the role of CCR5 in hepatocytes under NAFLD. Hepatocytes are the major liver parenchymal cells and are known to express functional CCR5 [9]. The protein interaction STRING database also showed direct protein interaction between human CCR5 and STAT3 proteins (Figure S1). These findings prompted us to study the regulation of STAT3 signaling in response to CCR5 activation in murine hepatocytes. To investigate the causal relationship between CCR5 deficiency and STAT3 signaling in primary hepatocytes, we harnessed a two-step collagenase perfusion technique [35] to isolate primary hepatocytes with high purity, high viability, and characteristic morphology (Figure 5a). To specifically determine the effect of ligand-induced CCR5 activation, primary hepatocytes were stimulated with CCL5 without or with pretreatment of CCR5 antagonist Maraviroc or STAT3 inhibitor Stattic (Figure 5b). CCL5 reduced STAT3 protein expression and the attenuation was fully reversed by Maraviroc. As expected, pretreatment with Stattic reduced STAT3 phosphorylation in primary hepatocytes.

### 2.8. CCR5 Regulates Hepatocyte Lipid Metabolism through STAT3

We examined the effect of CCL5 on lipid metabolism in primary hepatocytes by analyzing the expression levels of associated genes. As shown in Figure 5c, the expression of genes involved in free fatty acid uptake (*Cd36*) and de novo lipogenesis (*Acc-1* and *Scd-1*) was significantly reduced in CCL5-treated hepatocytes. Reversal of this attenuation by pretreatment with Maraviroc validated the contribution of CCR5. It is also consistent with exacerbated steatosis observed in mice with global CCR5 deficiency (Figure 1). On the other hand, pretreatment with Stattic further reduced the transcript levels of all three genes. Together with Figure 5b, these results demonstrated a causal relationship between STAT3 signaling and de novo lipogenesis in hepatocytes. Moreover, STAT3 expression and phosphorylation positively correlated with the expression levels of *Ppar-γ*, *Pgc-1α*, and *Pgc-1β* (Figure 5d). These results suggest that CCR5 signaling may mediate hepatocyte lipid metabolism through down-regulating STAT3 signaling. It should be noted that, even though CCR5 expression was gradually reduced in primary hepatocytes during ex vivo culturing (not shown), CCL5 and MVC treatment still had significant effects. In other words, CCR5 may play a more critical role than what we expected in regulating hepatocyte biology in vivo during NAFLD.

### 2.9. Blockade of CCR5 Signaling in g-MDSCs Reduces Hepatocyte STAT3 Signaling under the Fatty Liver Microenvironment

**Figure 6:**
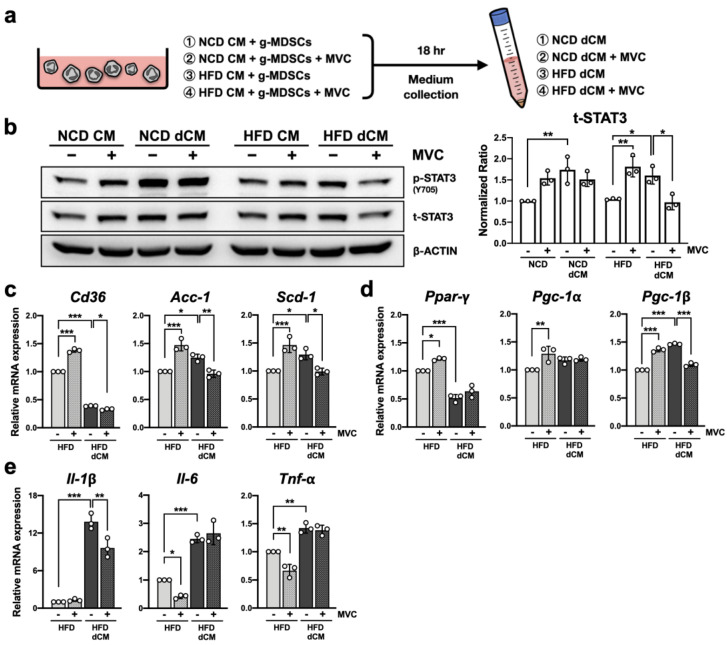
Blockade of g-MDSCs’ CCR5 signaling reduces STAT3 signaling and lipogenic gene expression in hepatocytes under the fatty liver microenvironment. (**a**) A schematic diagram of preparing g-MDSC-conditioned culture medium (doubly conditioned medium; dCM). (**b**) Primary WT hepatocytes were pretreated with 5 μM MVC (+) or vehicle control (−) for 1 h and then stimulated with liver-CM or g-MDSC-dCM for 18 h. Total lysates were analyzed by immunoblotting to determine the status of STAT3 expression. The amount of total STAT3 was normalized to β-ACTIN and set the first group as 1 (n = 3). The levels of transcripts from genes related to hepatic lipogenesis (**c**), *Ppar-γ* with its co-activators (**d**), and pro-inflammatory cytokines (**e**) were determined by real-time PCR. Expression was normalized to the *36b4* reference gene and further normalized to the average value of the first group (n = 3). *, *p* < 0.05; **, *p* < 0.005; ***, *p* < 0.001.

Since CCR5 signaling has been demonstrated to regulate the anti-inflammatory function of g-MDSCs (Figure 4) and lipid metabolism in primary hepatocytes (Figure 5), it is interesting to identify the role of CCR5 in the crosstalk between g-MDSCs and hepatocytes. To understand the profound secretomic influence of hepatic g-MDSCs on hepatocytes, we collected g-MDSC-conditioned liver-CM (doubly conditioned medium; dCM) to stimulate primary hepatocytes. WT g-MDSCs were cultured with either NCD-CM or HFD-CM with or without Maraviroc for 18 h, and supernatants were harvested to treat WT primary hepatocytes (Figure 6a). In the healthy liver microenvironment, g-MDSCs promoted STAT3 expression (Figure 6b, compare bars 1 and 3). Blockade of CCR5 signaling did not alter g-MDSCs’ ability in elevating STAT3 in hepatocytes (Figure 6b, compare bars 3 and 4). Intriguingly, in the NAFLD microenvironment, although g-MDSCs up-regulated STAT3 expression (Figure 6b, compare bars 5 and 7), null of CCR5 signaling strongly attenuated g-MDSCs’ capability of enhancing STAT3 signaling in hepatocytes (Figure 6b, compare bars 7 and 8).

### 2.10. Blockade of CCR5 Signaling in g-MDSCs Reduces Lipogenic Gene Expression in Hepatocytes under the Fatty Liver Microenvironment

To further investigate the alteration of lipid-metabolic and pro-inflammatory gene expression in hepatocytes, mRNA levels of hepatocytes stimulated with HFD-CM and HFD-dCM were analyzed. Consistent with data from R5-KO mice (Figure 3d), ex vivo blockade of CCR5 in NAFLD microenvironment elevated lipogenic gene expression (Figure 6c and 6d, compare bars 1 and 2). Together with Figure 5, these results showed that the presence or absence of CCR5 signaling strongly influenced lipogenic gene expression in hepatocytes. In addition, the same treatment reduced the expression of two major hepatic inflammatory genes, *Il-6* and *Tnf-α*, in hepatocytes (Figure 6e, compare bars 1 and 2).

Although g-MDSC-conditioned dCM slightly increased *Scd-1* and *Pgc-1β* expression in hepatocytes, the reduction of *Cd36* and *Ppar-γ* was even more significant (Figure 6c,d, compare bars 1 and 3). In addition, g-MDSC-conditioned dCM also greatly induced pro-inflammatory gene expression in hepatocytes (Figure 6e, compare bars 1 and 3). Notably, blockade of CCR5 on g-MDSCs considerably attenuated the expression of lipogenic genes, such as *Cd36*, *Acc-1*, *Scd-1*, and *Pgc-1β* (Figure 6c,d, compare bars 3 and 4), and the pro-inflammatory gene *Il-1β* (Figure 6e, compare bar 3 and 4), in hepatocytes under NAFLD microenvironment. These results led us to hypothesize that blockade of CCR5 signaling on g-MDSCs may reduce lipogenic and, to some extent, pro-inflammatory gene expression in hepatocytes under the condition of NAFLD.

### 2.11. CCR5-Deficient g-MDSCs Reduce Hepatic STAT3 Activation and Lipogenic Gene Expression in NAFLD

**Figure 7:**
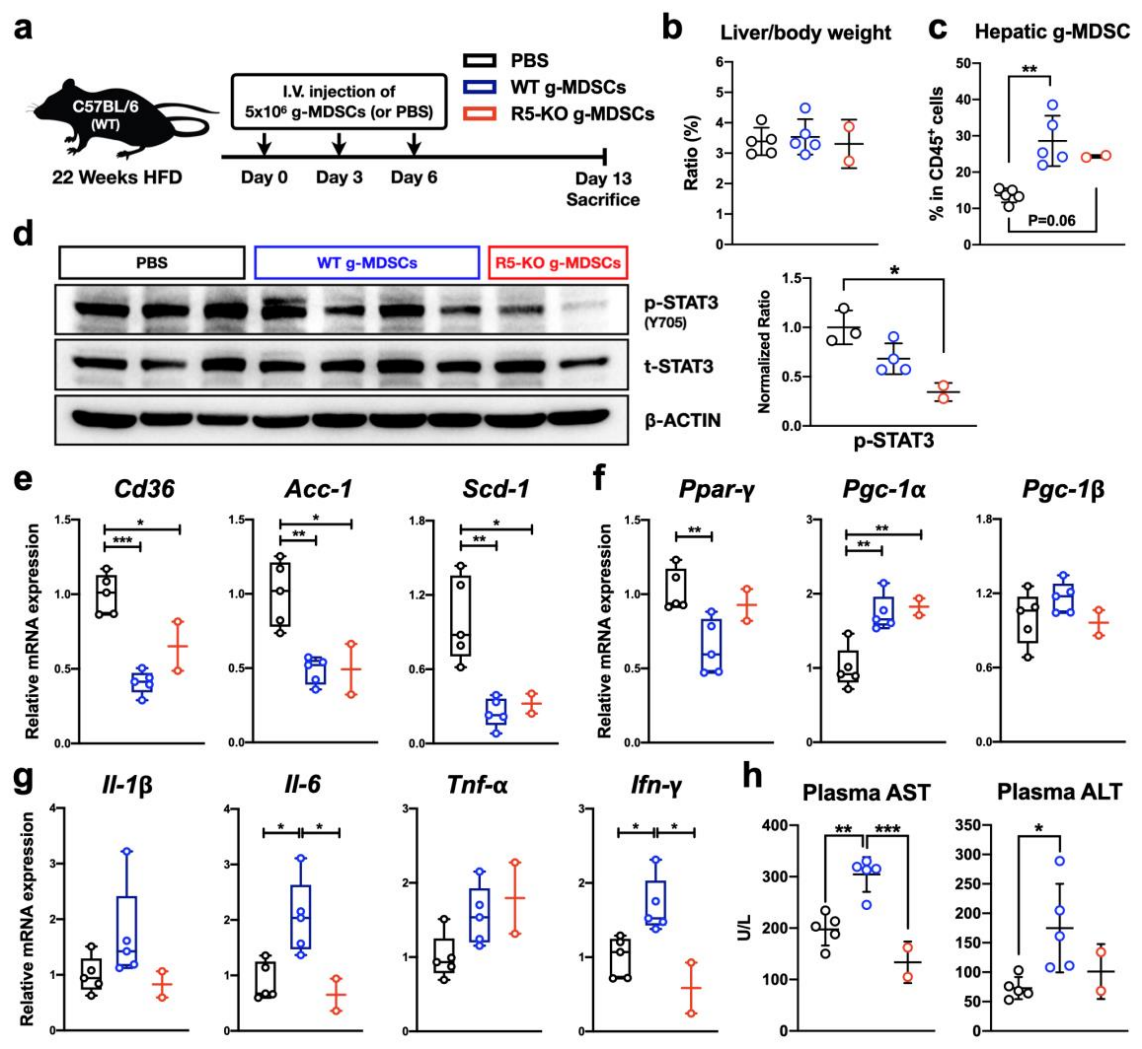
CCR5-deficient g-MDSCs reduce hepatic STAT3 activation and lipogenic gene expression in NAFLD. (**a**) A schematic diagram of the animal model for g-MDSC adoptive transfer. Obese WT mice were retro-orbitally injected with PBS, WT g-MDSCs, or R5-KO g-MDSCs three times before being sacrificed by 24 weeks of HFD. (**b**) The liver-to-body weight ratio was measured and calculated. (**c**) The amount of hepatic g-MDSCs was quantitated by flow cytometry and shown as percentages in the hematopoietic population as described for Figure 2. (**d**) The status of STAT3 phosphorylation was determined by immunoblotting of liver homogenates. The signal of phosphorylated STAT3 was normalized to total STAT3 and set the average value of the PBS-injected group to 1. Hepatic transcript levels of genes related to lipogenesis (**e**), *Ppar-γ* with its co-activators (**f**), and pro-inflammatory cytokines (**g**) were determined by real-time PCR. Expression was normalized to *the 36b4* reference gene and further normalized to the average value of the PBS-injected group. (**h**) Plasma concentrations of AST and ALT were analyzed (n = 2-5). *, *p* < 0.05; **, *p* < 0.005; ***, *p* < 0.001.

To test our hypothesis, we conducted an adoptive transfer of freshly isolated g-MDSCs into WT obese mice three times before sacrifice (Figure 7a). Adoptive transfer of WT or R5-KO g-MDSCs did not alter the liver-to-body weight ratio of WT obese mice (Figure 7b). Nevertheless, the percentage of g-MDSCs among leukocytes in the liver was elevated in mice receiving g-MDSC transfer (Figure 7c). Alterations of hepatic signaling profile and gene expression were also significant. Transferring R5-KO g-MDSCs substantially reduced hepatic STAT3 activation (Figure 7d). Consistent with the inhibitory effects of Maraviroc in our ex vivo assay using g-MDSC-conditioned dCM (Figure 6b), hepatic STAT3 activation status was reduced in mice receiving R5-KO g-MDSCs in comparison with WT g-MDSCs (Figure 7d).

A consistent pattern of gene expression downstream of STAT3 signaling was also observed. As compared to the phosphate-buffered saline (PBS) control group, adoptive transfer of both WT and R5-KO g-MDSCs led to a significant decrease of three genes associated with hepatic de novo lipogenesis (Figure 7e) and an increase of fatty acid oxidation-related gene *Pgc-1α* (Figure 7f). On the other hand, the expression of pro-inflammatory cytokines, such as *Il-1β*, *Il-6,* and *Ifn-γ*, was elevated in obese WT mice receiving WT g-MDSCs, but not R5-KO g-MDSCs (Figure 7g). More importantly, even though the adoptive transfer of WT g-MDSCs elevated the levels of liver damage plasma markers, the adoptive transfer of R5-KO g-MDSCs did not increase plasma AST and ALT compared with PBS control (Figure 7h). This is consistent with the pattern of pro-inflammatory cytokines in the NAFLD microenvironment (Figure 7g). Therefore, our results suggest that the adoptive transfer of CCR5-deficient g-MDSCs can reduce hepatic lipogenic gene expression without exacerbating inflammatory responses and liver damage.

## 3. Discussion

Given that CCR5 is pivotal for facilitating immune responses, potential NAFLD treatments targeting CCR5 have been investigated. Previous studies from Blanco’s group demonstrated that a diet incorporating CCR5 antagonist Maraviroc significantly reduced hepatic triglyceride accumulation and steatosis under diet-induced NAFLD [13,14]. Nevertheless, our data unexpectedly revealed that global CCR5 deficiency exacerbated steatosis in a murine NAFLD model. These contradictory results may be explained by their virtue of targeting different mechanisms. Our NAFLD model using genetic deletion of *Ccr5* aimed to describe the result of a systemic defect of CCR5, whereas pharmacological inhibition of CCR5 through diet might primarily affect the dynamics of microbiota in the gastrointestinal tract. In fact, the liver is a critical nutrition hub as it gathers nutrition flow drained from the gut via the portal vein. As Blanco’s group described, intake of Maraviroc significantly modified gut microbiota composition in their NAFLD model [36]. Ameliorated hepatic steatosis might result from altered microbiota composition via Maraviroc treatment but not from CCR5 blockade in the systemic circulation.

In our experimental condition, CCR5-KO mice exhibited more severe insulin resistance than WT mice under HFD feeding (not shown). Other investigations supporting our results of genetic CCR5 deletion are from Hasty’s and Hong’s reports demonstrating that CCR5 knockout in the genetic background led to severe systemic glucose intolerance in NAFLD [37] and exacerbated steatosis in alcoholic fatty liver disease [15]. Nevertheless, Ota’s group suggested the polar opposite results in which they found CCR5-deficient mice were protected from insulin resistance, hepatic steatosis, and diabetes induced by 10 weeks of HFD (60% calories from fat) starting at the age of 5 weeks [38]. Some possible explanations include differences in HFD recipe, diet duration, and source of knockout mice. It is also plausible that CCR5 plays a different role in different stages of NAFLD.

It is widely accepted that CCR5 signaling is pivotal in mediating hepatic lipid accumulation in diet-induced obesity. Therefore, understanding the molecular mechanism of how CCR5 regulates lipid metabolism becomes increasingly important to discover novel therapeutic targets. Peroxisome proliferator-activated receptors (PPARs) are critical nuclear receptors that regulate glucose and lipid metabolism in NAFLD [39]. Although PPARα, one of three PPAR isoforms, is most highly expressed in the liver, CCR5 deficiency did not alter hepatic *Ppar-α* expression in NAFLD (Figure S2). On the other hand, hepatic expression of steatogenic *Ppar-γ* with its downstream lipogenic genes [40] was found susceptible to null of CCR5 signaling in our study. Aside from increased lipogenesis, dysfunction of mitochondrial fatty acid oxidation may also contribute to excessive lipid storage in NAFLD. Nevertheless, influences of CCR5 deficiency on hepatic β-oxidation-related genes, including *Cpt-1*, *Cpt-2*, *Acox-1*, *Scad*, *Mcad*, and *Lcad*, did not show a consistent pattern (Figure S2).

Conversely, hepatic pro-inflammatory cytokines, such as *Il-6*, *Il-1β*, *Ifn-γ*, and *Tnf-α*, were significantly reduced in CCR5-deficient obese mice (Figure S2). Expression of STAT3 activators, such as IL-6 and granulocyte-colony stimulating factor (G-CSF), is significantly elevated in the NAFLD microenvironment. Theoretically, CM prepared from fatty livers should capture these cytokines and mediate STAT3 responses. Indeed, our unbiased ex vivo liver-CM system, inspired by Male’s group [41], recaptured a similar CCR5-KO phenotype described above in primary hepatocytes under the NAFLD microenvironment. Most importantly, we showed that CCR5 modulated hepatic lipid metabolism by regulating STAT3 signal transduction. Interestingly, while the role of STAT3 signaling was described in several liver diseases [42], the impact of direct STAT3 down-regulation in primary hepatocytes is still undefined so far. Our results provide a glimpse of the relationship between STAT3 signaling and lipogenic gene expression in hepatocytes. Our data also suggest that CCR5 signaling may indirectly regulate STAT3 protein expression and phosphorylation in hepatocytes. A previous report showing that the blockade of CCR5 inhibits the IL-6-STAT3 pathway via up-regulating suppressor of cytokine signaling 3 (SOCS3) [43] is consistent with an indirect role of CCR5 in STAT3 regulation. Regardless, STAT3 signaling may be critical in connecting g-MDSCs and hepatocytes during NAFLD progression.

G-MDSCs and neutrophils share the same myeloid origin and are similar in many morphological and phenotypic features. However, g-MDSCs are specifically defined by their immunosuppressive activities [44]. Both g-MDSCs and neutrophils undergo maturation with environmental stimuli, such as G-CSF, granulocyte-macrophage-colony stimulating factor (GM-CSF), and IL-6, and express significant granules including myeloperoxidase (MPO) and neutrophil elastase (NE) during their maturation. Although the role of g-MDSCs in tumor-bearing sites is widely studied, their role in NAFLD is largely unclear. Enriched IL-6, G-CSF and GM-CSF expression in NAFLD microenvironment [45] may serve as a perfect milieu for facilitating the maturation and function of g-MDSCs. Indeed, our investigation showed that g-MDSCs had a significantly high survival rate, a high level of STAT3 activation, and expressed a large amount of *Mpo* and *Ne* (Figure S3) in the NAFLD microenvironment.

Although g-MDSCs might gain many activated neutrophil-like traits in fatty liver, they also expressed a great amount of critical immunosuppressive factors, such as *Il-10*, *Tgf-β,* and *Pd-l1*. G-MDSCs activate regulatory T cells by secreting IL-10 and TGF-β [16] and inactivate T cells by direct binding to PD-1 receptors via PD-L1 [46]. Interestingly, the blockade of CCR5 signaling on g-MDSCs reduced their *Il-10*, *Tgf-β,* and *Pd-l1* expression (Figure 4e). It suggests that CCR5 may be a key player in mediating g-MDSCs’ immunosuppressive activities in NAFLD. In the healthy liver scenario, the low survival rate might result from a low STAT3 signal in g-MDSCs, since STAT3 phosphorylation is responsible for the accumulation of MDSCs. Higher levels of ARG1 expressed by these relatively immature g-MDSCs in the healthy liver microenvironment (Figure 4d) suggest that they might suppress T cell activation through L-arginine deprivation. This immunosuppressive activity could be further enhanced by CCR5 inhibition. Direct measurement of T cell activity in the NAFLD microenvironment will further validate the immunosuppressive activity of hepatic g-MDSCs.

In this study, we specifically focused on resolving the direct influence of hepatic g-MDSCs on hepatocytes since aggregates of Gr-1 positive cells were observed physically adjacent to parenchymal hepatocytes in several NASH models [47]. Although less is known about g-MDSCs’ impact on hepatocytes, neutrophils can directly cause hepatocyte injury by secreting MPO in NASH [48]. Given that the MPO activity of g-MDSCs has been demonstrated to be greater than neutrophils’ [49], hepatic g-MDSC may be a critical immune cell population that contributes to liver damage. Our result surprisingly showed that CCR5 blockade significantly reduced the *Mpo* expression in g-MDSCs under the NAFLD microenvironment (Figure S3), suggesting a potential strategy to ameliorate liver damage targeting CCR5 on g-MDSCs. Additionally, the CCR5-blocked g-MDSC-conditioned medium greatly increased the expression of SOCS3 in hepatocytes (Figure S4, bar 4). As SOCS3 is a critical negative regulator of STAT3 [50], it may contribute to reduced STAT3 signaling in hepatocytes (Figure 6b).

Knowing that the STAT3 pathway is pivotal for lipid metabolism in hepatocytes (Figure 5), STAT3 signaling may be central in the relationship between g-MDSCs and hepatocytes. Our results from adoptive g-MDSC transfer further confirmed that CCR5-deficient g-MDSCs reduced hepatic STAT3 activation and lipogenic gene expression without exacerbating liver injury in NAFLD. Nevertheless, the liver mass to body weight ratio and liver damage markers of mice did not show significant reduction after 2 weeks of CCR5-deficient g-MDSC transfer compared with the PBS-injected group (Figure 7b,h). It suggests that our prototype of adoptive transfer of CCR5-deficient g-MDSCs as a new therapy for NAFLD should be further revised. Recently, several nanoparticle systems have been described to target MDSCs for drug delivery [51]. We, therefore, hypothesize that delivering Maraviroc to bone marrow g-MDSCs via nanoparticle techniques may alleviate steatosis in NAFLD. Similar research from Ma’s group suggests targeting bone marrow CCL5/CCR5 signaling via nanoparticle delivery is promising in modulating immature myeloid cells in their tumor model [25]. More studies will be needed to further evaluate the potential of applying an immunotherapeutic strategy in NAFLD.

## 4. Materials and Methods

### 4.1. Animal Studies

Male C57BL/6 (WT) mice were purchased from BioLASCO (Taipei, Taiwan). Male R5-KO mice Ccr5tm1Kuz/J were purchased from the Jackson laboratory (Bar Harbor, ME). Breeding of WT and R5-KO mice was carried out in the AAALAC-certified animal facility at Chang Gung University. WT and R5-KO mice were backcrossed at least ten times to synchronize their genetic background. For each mouse model, mice were randomly assigned into two groups and fed with HFD (45% calories from fat) or NCD (13.5% calories from fat) based on experimental design. Body weight, food, and water intake were recorded weekly.

### 4.2. Biochemical Analysis of Blood and Liver Samples

Blood samples were obtained by retro-orbital bleeding under anesthesia. Separation of plasma was performed by centrifugation at 9300× *g* for 10 min at 4 °C and stored at −80 °C before analysis. Plasma levels of ALT, AST, and ALP were analyzed by Bio-Cando Co. (Taipei, Taiwan). Immediately after removal from mice, parts of liver samples were collected and snap-frozen in liquid nitrogen. Hepatic lipids were extracted by homogenizing 20 mg of liver tissue in 1 mL of 5% Triton-X100, and heated to 80 °C for 5 min. The samples were cooled down and heated again to fully solubilize all lipids. After centrifugation for 5 min, supernatants were diluted for further detection. Hepatic triglyceride level was determined by a colorimetric assay kit (Randox, Crumlin, UK) in accordance with the manufacturer’s protocols.

### 4.3. Liver Sectioning and Staining

Parts of liver tissues were fixed overnight in a neutral-buffered formalin solution. Tissue embedding and sectioning were performed by Bio-Cando Co. (Taipei, Taiwan). Paraffin sections were processed for hematoxylin-eosin staining and frozen sections were processed for Oil-red O staining using standard procedures. Stained liver sections were examined using the Zeiss Axio Imager M2 bright field microscope and 200×magnification. Images were captured with the Nikon D5100 digital camera.

### 4.4. Profiling of Immune Cells Infiltrated in Hepatic Tissue

Part of freshly excised liver tissue was digested with type IV collagenase (Gibco) and then mashed through a 100 μm cell strainer (Falcon). Hepatocytes and cell debris were removed by centrifugation at 20 x *g* for 5 min at room temperature twice. After red blood cell lysis, cells were subjected to Fc blocking (Purified Rat Anti-Mouse CD16/CD32, BD Biosciences) for 15 min at room temperature before antibody staining. Cells isolated from 60 mg of liver tissue were resuspended in 200 μL of PBS supplemented with 2% fetal bovine serum (FBS) in a 5 mL polystyrene round-bottom tube (Falcon). For surface staining, cells were stained with specific antibodies for 15 min at room temperature in the dark. FVS780 (Cat# 565388), PerCP-Cy5.5-conjugated CD45.2 (104, Cat#552950), BB515-conjugated CD11b (M1/70, Cat#564454), BV510-conjugated Gr-1 (RB6-8C5, Cat# 563040), PE-Cy7-conjugated Ly-6C (AL-21, Cat# 560593), and APC-conjugated Ly-6G (1A8, Cat# 560599) were from BD Biosciences. All stained cells were analyzed using an Attune NxT Flow Cytometer (Thermo Fisher Scientific, Waltham, MA, USA). Acquired data were further analyzed via FlowJo (Tree Star, Inc., Ashland, OR, USA).

### 4.5. Liver-Conditioned Medium (CM) for Ex Vivo Experiments

Liver-CM was generated by culturing 1 g of finely minced liver tissue in 2 mL of serum-free M199 medium (GeneDireX) for 48 h. Cell debris was removed by passing through a 100 μm cell strainer (Falcon), followed by centrifugation at 500× *g* for 5 min. The protein concentration of the conditioned medium was measured by Pierce BCA Protein Assay Kit (Thermo Fisher Scientific) and then normalized. Primary g-MDSCs and hepatocytes were stimulated with 10% *v/v* liver-CM for the indicated time.

### 4.6. Isolation and Culturing of Primary Hepatocytes and g-MDSCs

A rapid two-step method was used to isolate primary hepatocytes. Briefly, EDTA-containing perfusion buffer and digestion buffer with collagenase (100 U/mL, Sigma type IV) were pumped into the liver after portal vein cannulation. Mincing the livers and filtering through cotton gauze liberated the cells. The hepatocytes were purified from non-parenchymal cells and nonviable hepatocytes by Percoll density gradient centrifugation at 200× *g*. Primary hepatocytes were then counted and plated in a collagen-coated culture plate with DMEM plating media containing 10% FBS. Three hours after plating, plating media were replaced by maintenance media (1:1 mixture of DMEM and Ham’s F-12 with l-glutamine), supplemented with 10% FBS, 500 nM dexamethasone, 2 μg/mL of insulin, 1 μg/mL of transferrin, and 1.34 ng/mL of selenite (R&D Systems). Primary MDSCs were freshly isolated from mouse bone marrow in tibias and femurs, followed by staining with MDSC-specific surface markers. Live g-MDSCs were identified by staining with FVS780, BB515-conjugated CD11b, PE-Cy7-conjugated Ly-6C, and APC-conjugated Ly-6G antibodies. A distinct cell population was collected by fluorescence-activated cell sorting (FACS) in FACSAria IIu Sorter (BD Biosciences). Purified g-MDSCs were cultured in RPMI supplemented with 10% heat-inactivated FBS and antibiotics. For ex vivo experiments, cells were seeded in a multi-well plate and treated with the following reagents: 10 ng/mL of CCL5 (ProSpec), 5 μM Maraviroc (AdooQ Bioscience), and 10 μM Stattic (AdooQ Bioscience). Images were taken using the ZOE Fluorescent Cell Imager (Bio-Rad) under bright field.

### 4.7. Immunoblotting

Samples were first homogenized in RIPA lysis buffer with PMSF (OmniPur, Merck Millipore) and protein phosphatase inhibitor cocktail (BIONOVAS Biotechnology). Cleared protein samples were subjected to SDS-PAGE, transferred to a nitrocellulose membrane, blocked by protein-free blocking buffer (BM01-500, Visual Protein), and then blotted with primary antibodies. The antibody for tyrosine-phosphorylated STAT3 (Cat# 9131) was from Cell Signaling Technology. The antibody for STAT3 (H-190, Cat# sc-7179) was from Santa Cruz Biotechnology. Normalization was confirmed by β-ACTIN immunoblot. The antibody for β-ACTIN (2D4H5, Cat# 66009-1-Ig) was from Proteintech. Signals were visualized with HRP-conjugated anti-mouse IgG (Santa Cruz Biotech) or anti-rabbit IgG (Jackson ImmunoResearch), followed by enhanced chemiluminescence reaction and detection in the BioRad ChemiDoc Touch Imaging System. Band intensities were quantified using ImageJ (NIH, MD).

### 4.8. Tissue RNA Analysis by Quantitative RT-PCR

Total RNAs were prepared using TRIzol Reagent (Invitrogen) according to the manufacturer’s instructions. RNA quality and quantity were determined using NanoDrop 2000 (Thermo Scientific) before cDNA synthesis. Quantitative PCR was carried out using the BioRad CFX Connect Real-Time System with SYBR-Green Master Mix according to the manufacturer’s protocol. Reactions were run in duplicates using *36b4* as a reference gene. Primer sequences were listed in Table 1.

### 4.9. Statistical Analysis

The statistical significance of differences between groups was analyzed and illustrated with GraphPad Prism by performing the indicated statistical tests. Potential outliers were determined by Grubb’s test (*p* < 0.05) using a free calculator on the GraphPad site and excluded from the statistical analyses. Data were expressed as mean ± standard deviation (SD) from at least three independent experiments. The difference was analyzed by one-way analysis of variance (ANOVA) followed by Tukey’s post hoc tests for multiple comparisons. The significant difference was set at *p* < 0.05.

## 5. Conclusions

**Figure 8:**
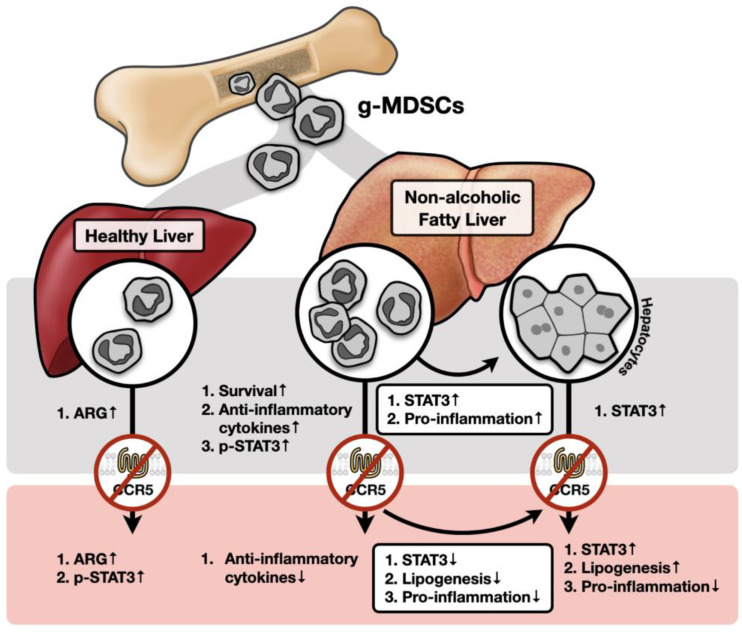
A schematic diagram of key conclusions. Results from our mechanistic studies of NAFLD in WT (gray area) and CCR5-deficient (pink area) backgrounds are illustrated in the proposed diagram.

Summarizing our ex vivo results, we proposed a model diagrammed in Figure 8. When myeloid g-MDSCs egress from bone marrow and travel to a healthy hepatic microenvironment, they produce a high amount of ARG1 as their main immunosuppressive function, which will be further enhanced by the blockade of CCR5 signaling. In a fatty liver scenario, g-MDSC possesses a higher survival rate and produces other anti-inflammatory factors, such as *Il-10*, *Tgf-β*, and *Pd-l1*, instead of *Arg*. Furthermore, while g-MDSCs enhance hepatocytes’ expression of pro-inflammatory cytokines in the NAFLD microenvironment, blockade of CCR5 surprisingly decreases g-MDSC-induced STAT3 phosphorylation, lipogenic gene expression, and *Il-1β* expression in hepatocytes. Experiments of adoptive g-MDSC transfer further confirmed that CCR5-deficient g-MDSCs reduced hepatic STAT3 signaling, lipogenic gene expression, and pro-inflammatory gene expression. These results suggest a potential therapeutic strategy for NAFLD.

## Figures and Tables

**Table 1:** Primer sequences for real-time RT-PCR analysis.

Gene Symbol	Forward Primer (5′-3′)	Reverse Primer (5′-3′)
*36b4*	CGACCTGGAAGTCCAACTAC	ATCTGCTGCATCTGCTTG
*Ccr5*	TTTTCAAGGGTCAGTTCCGAC	GGAAGACCATCATGTTACCCAC
*Arg-* *1*	AACACGGCAGTGGCTTTAACC	GGTTTTCATGTGGCGCATTC
*Tgf-* *β*	TGACGTCACTGGAGTTGTACGG	GGTTCATGTCATGGATGGTGC
*Pd-l1*	GACCAGCTTTTGAAGGGAAATG	CTGGTTGATTTTGCGGTATGG
*Il-* *10*	CAGAGCCACATGCTCCTAGA	TGTCCAGCTGGTCCTTTGTT
*Il-* *6*	TACCACTTCACAAGTCGGAGGC	CTGCAAGTGCATCATCGTTGTTC
*Tnf-* *α*	GCCTCTTCTCATTCCTGCTTG	CTGATGAGAGGGAGGCCATT
*Il-* *1β*	CCTTCCAGGATGAGGACATGA	TGAGTCACAGAGGATGGGCTC
*Ifn-* *γ*	GCCATCAGCAACAACATAAGCGTC	CCACTCGGATGAGCTCATTGAATG
*Cd36*	TCCTCTGACATTTGCAGGTCTATC	AAAGGCATTGGCTGGAAGAA
*Acc-* *1*	AGGAAGATGGCGTCCGCTCTG	GGTGAGATGTGCTGGGTCAT
*Scd-* *1*	CCGGAGACCCTTAGATCGA	TAGCCTGTAAAAGATTTCTGCAAA
*Ppar-* *γ*	CGGTTTCAGAAGTGCCTTG	GGTTCAGCTGGTCGATATCAC
*Pgc-* *1α*	ACTGAGCTACCCTTGGGATG	TAAGGATTTCGGTGGTGACA
*Pgc-* *1β*	TCCAGAAGTCAGCGGCCTTGTGTCA	CTCTGGGACAGGGCAGCACCGA

## Data Availability

Not applicable.

## Supplementary Materials

The following supporting information can be downloaded at: https://www.mdpi.com/article/10.3390/ijms232113048/s1, Figure S1: Protein interaction analysis of human CCR5; Figure S2: Gene expression profile of liver tissues from lean and obese WT versus R5-KO mice; Figure S3: Fatty liver microenvironment promotes g-MDSC maturation; Figure S4: Distinct expression pattern of SOCS family members in the fatty liver microenvironment.
