# Supplementary material for: Granulocytic MDSC with Deficient CCR5 Alleviates Lipogenesis and Inflammation in Nonalcoholic Fatty Liver Disease
Source: Int J Mol Sci. 2022 Oct 27;23(21):13048. doi: 10.3390/ijms232113048 (PMC9656569; doi:10.3390/ijms232113048)
Supplement: Supplementary file 1 [file ijms-23-13048-s001.zip › ijms-1906588-supplementary.pdf]

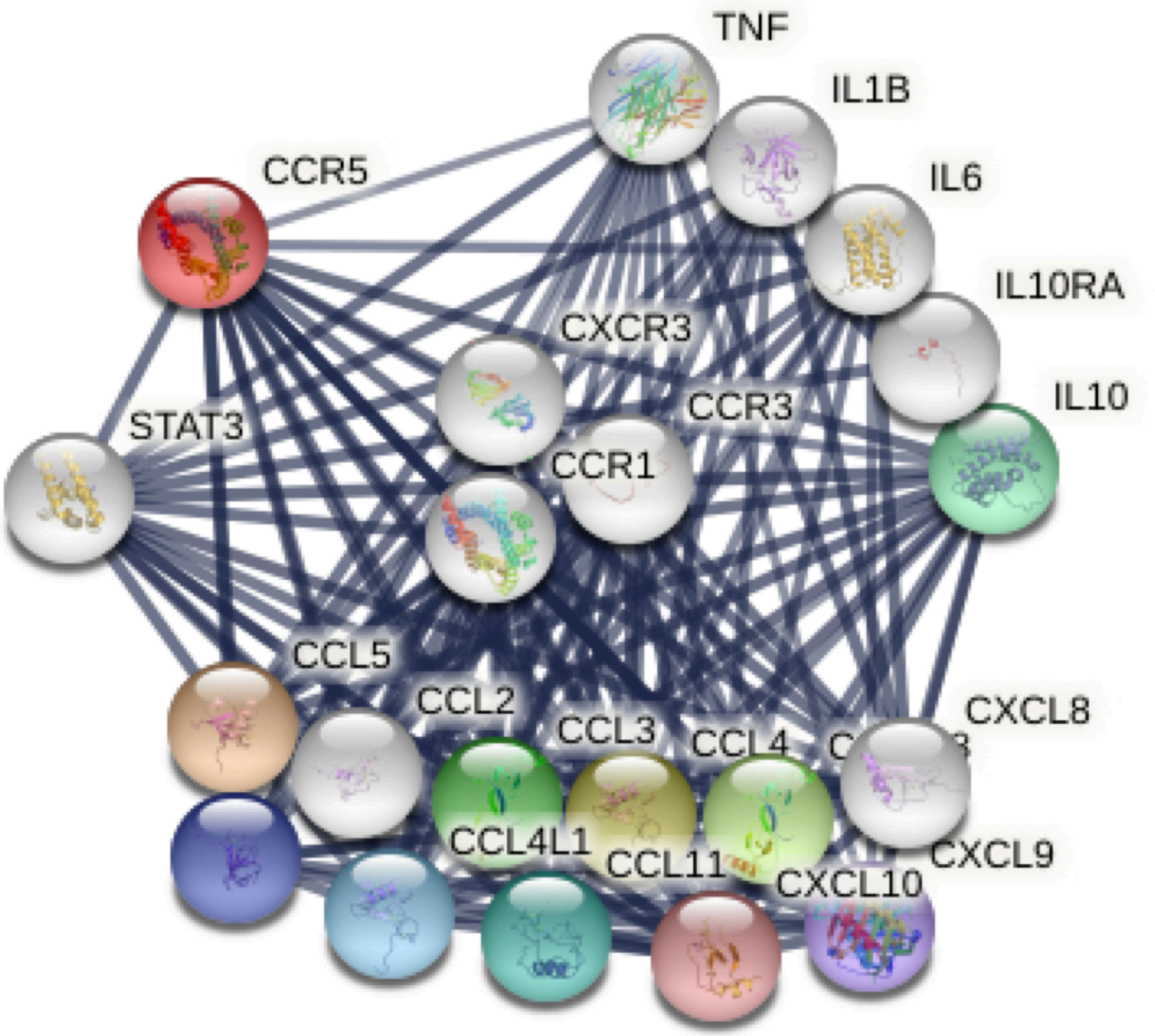


**Figure S1.** Protein interaction analysis of human CCR5. The schematic network was constructed by the STRING database version 11.0. This network identifies 20 *Homo sapiens* CCR5 interacting partners based on previously reported evidence.

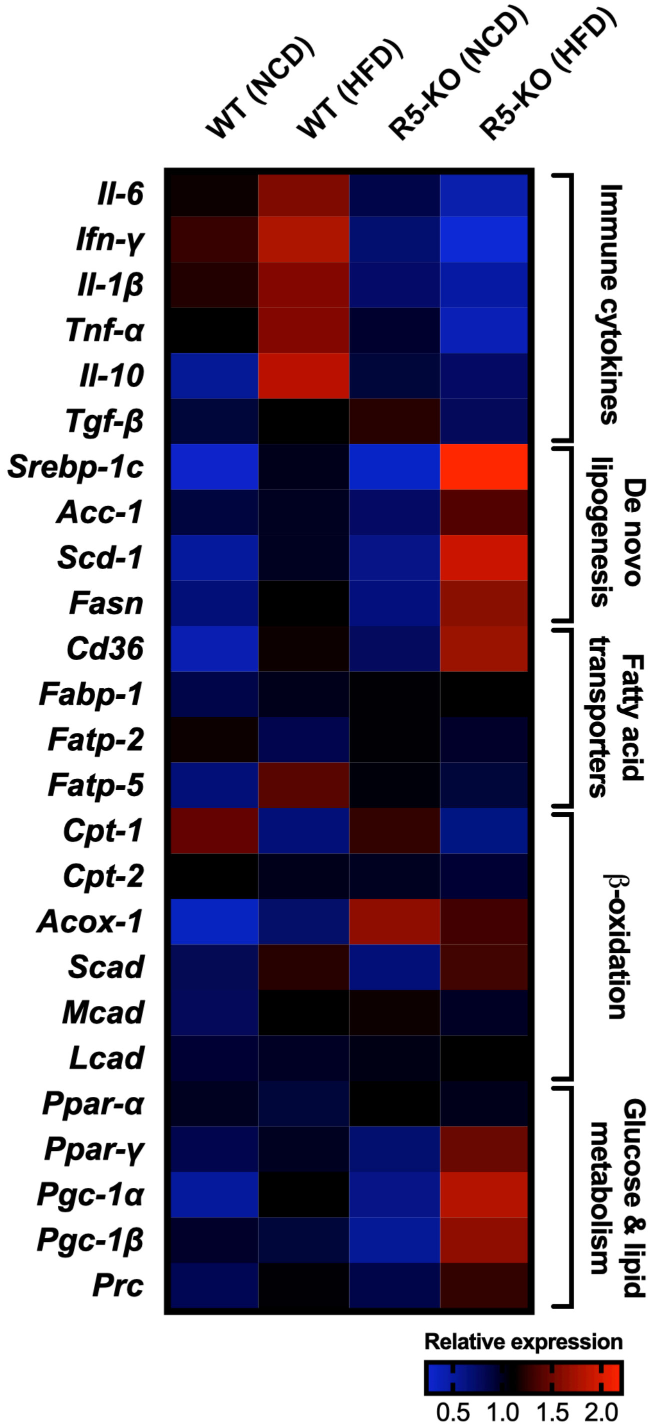


**Figure S2.** Gene expression profile of liver tissues from lean and obese WT versus R5-KO mice. Expression of 25 genes were analyzed by real-time PCR as described for Fig. 3 and their relative expression level was presented as a heat map. Functional groups of these genes are denoted on the right.

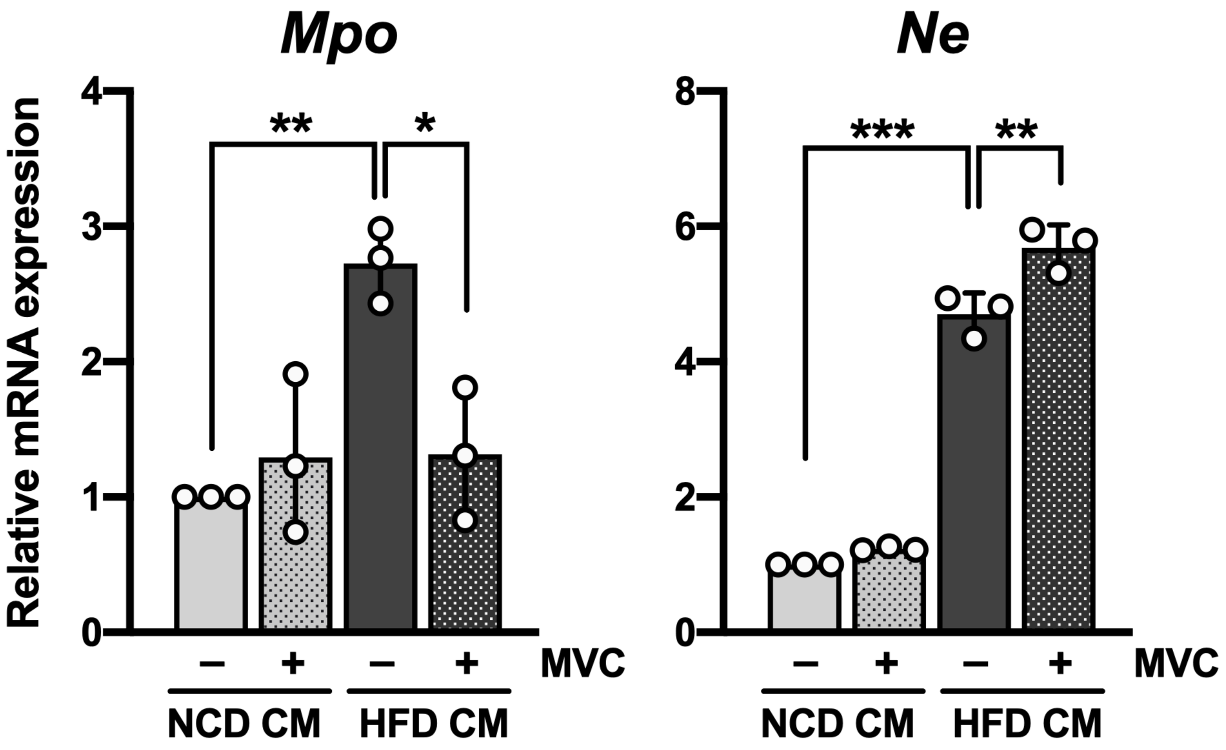


**Figure S3**. Fatty liver microenvironment promotes g-MDSC maturation. Primary g-MDSCs from WT mice were treated with liver-conditioned media in the presence (+) or absence (-) of MVC. The transcript levels of *Mpo* and *Ne* were analyzed as described for Fig. 4.

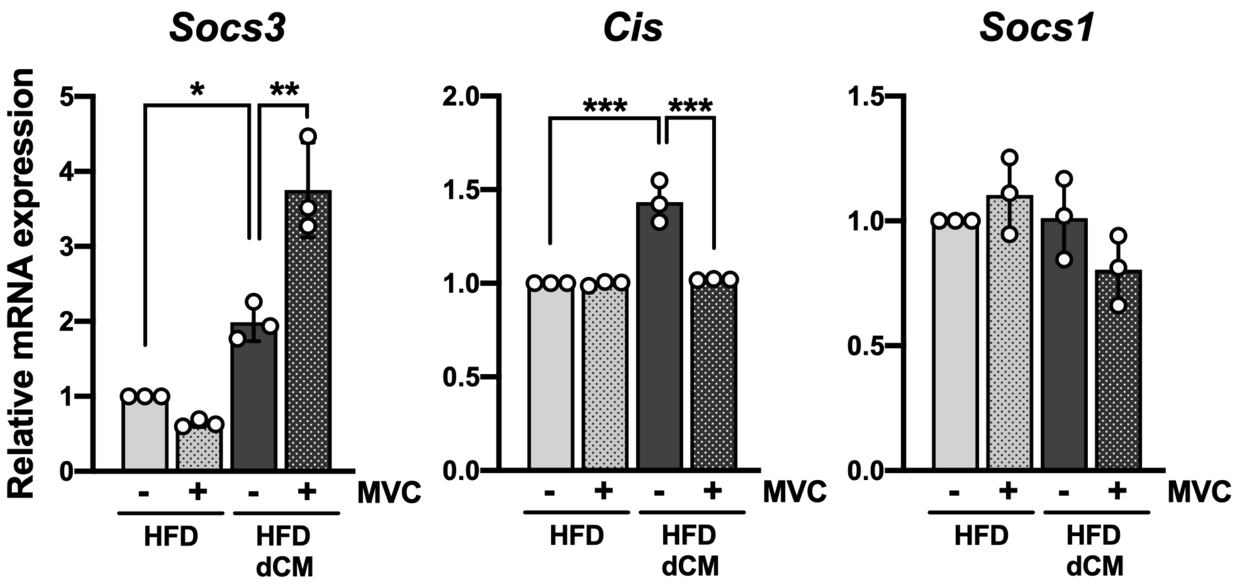


**Figure S4.** Distinct expression pattern of SOCS family members in fatty liver microenvironment. Primary g-MDSCs from WT mice were treated with liver-conditioned media in the presence (+) or absence (-) of MVC. The transcript levels of *Socs3*, *Cis* and *Socs1* were analyzed as described for Fig. 4.
